# Synergy between EngE, XynA and ManA from *Clostridium cellulovorans* on corn stalk, grass and pineapple pulp substrates

**DOI:** 10.1007/s13205-011-0011-y

**Published:** 2011-06-18

**Authors:** B. Olver, J. S. Van Dyk, N. Beukes, B. I. Pletschke

**Affiliations:** Department of Biochemistry, Microbiology and Biotechnology, Rhodes University, Grahamstown, 6139 South Africa

**Keywords:** Corn stalk, EngE, Grass, ManA, Pineapple pulp, XynA

## Abstract

The synergistic interaction between various hemi/cellulolytic enzymes has become more important in order to achieve effective and optimal degradation of complex lignocellulose substrates for biofuel production. This study investigated the synergistic effect of three enzymes endoglucanase (EngE), mannanase (ManA) and xylanase (XynA) on the degradation of corn stalk, grass, and pineapple fruit pulp and determined the optimal degree of synergy between combinations of these enzymes. It was established that EngE was essential for degradation of all of the substrates, while the hemicellulases were able to contribute in a synergistic fashion to increase the activity on these substrates. Maximum specific activity and degree of synergy on the corn stalk and grass was found with EngE:XynA in a ratio of 75:25%, with a specific activity of 41.1 U/mg protein and a degree of synergy of 6.3 for corn stalk, and 44.1 U/mg protein and 3.4 for grass, respectively. The pineapple fruit pulp was optimally digested using a ManA:EngE combination in a 50:50% ratio; the specific activity and degree of synergy achieved were 52.4 U/mg protein and 2.7, respectively. This study highlights the importance of hemicellulases for the synergistic degradation of complex lignocellulose. The inclusion of a mannanase in an enzyme consortium for biomass degradation should be examined further as this study suggests that it may play an important, although mostly overlooked, role in the synergistic saccharification of lignocellulose.

## Introduction

Lignocellulose consists of approximately 75% carbohydrates which makes it an ideal feedstock for biofuel production (Bayer et al. 2007; Lynd et al. 1991). A whole range of enzymes is required to work in synergy to degrade these substrates. Synergy is established when the combined action of enzymes is greater than the sum of the individual activities on the same substrate. While synergy between cellulases on cellulose has been well studied (Andersen et al. 2008; Boisset et al. 2001; Teeri 1997; Woodward 1991) the synergy between cellulases and hemicellulases and between hemicellulases on their own has only recently garnered more attention (Banerjee et al. 2010; Gao et al. 2010; Prior and Day 2008; Selig et al. 2009). This is most probably because the main focus in biofuel production has been the production of glucose which is the optimal substrate for ethanol fermentation in prominent fermentation organisms such as *Saccharomyces cerevisiae* (Himmel et al. 2007). However, ignoring the hemicellulose fraction of a lignocellulose substrate for eventual fermentation reduces the total yield of sugars obtainable (Merino and Cherry 2007). The hemicellulose fraction and its saccharification have therefore gained prominence even though it may only form a small percentage of the carbohydrates in the substrate. Thus, hemicellulases such as xylanases and mannanases are increasingly being investigated for their role in enhancing the saccharification of substrates and increasing the overall yield of sugars obtained.

The enzymes in this study are all cellulosomal enzymes derived from *Clostridium cellulovorans*. EngE is a glycosyl hydrolase family 5 endoglucanase and is one of the three major subunits of the *C. cellulovorans* cellulosome (Tamaru and Doi 1999). XynA is a family 11 glycoside hydrolase from the cellulosome of *C. cellulovorans* (Kosugi et al. 2002). XynA also contains an acetyl xylan esterase module that contributes to deacetylation of acetylated substrates and displays a synergy with the endo-xylanase catalytic domain (Kosugi et al. 2002). ManA is a cellulosomal enzyme from *C. cellulovorans* and is classified as a family 5 glycosyl hydrolase (Tamaru and Doi 2000). EngE and ManA have very narrow substrate specificities and are only able to cleave specific linkages and are therefore not cross-reactive (Tamaru and Doi 1999; 2000). In this study, we evaluated the interactions between these three enzymes for their synergistic effects on three complex substrates.

The lignocellulosic substrates in this study were corn stalk (or corn stover), kikuyu grass and pineapple fruit pulp. Corn stover contains approximately 41.7% cellulose, 20% hemicellulose and 17% lignin and is considered one of the main lignocellulose substrates for biomass conversion in the USA (Merino and Cherry 2007). The compositions of different grasses vary and, for bermuda grass, has been reported as containing 47.8% cellulose, 13.3% hemicellulose and 19.4% lignin (Li et al. 2009), while verge grass contains 30.7% cellulose, 15.6% hemicellulose and 14.1% lignin (Dijkerman et al. 1997). Switchgrass contains 31% cellulose, 22% hemicellulose and 18% lignin (Merino and Cherry 2007). No specific composition has been reported for the grass used in this study, kikuyu (*Pennisetum clandestinum* or *Cenchrus clandestinus*) from the Poaceae family. However, it may be able to serve as a possible municipal waste product for lignocellulose bioconversion as it is extensively used on sports fields and domestic gardens. Pineapple fruit pulp is a waste product from the fruit juice industry which remains after removal of the juice. It is a waste product that is generated in large quantities in the juice industry, and degradation using hemi/cellulolytic enzymes may generate useful bioproducts such as sugars for biofuel production. Ban-koffi and Han (1990) indicated that the composition of pineapple waste was 19% cellulose, 22% hemicellulose, 5% lignin and 53% cell soluble matter.

While most hemicellulose contains a large component of xylan, the mannan composition is generally ignored and virtually no research has been carried out with respect to mannan degradation and the extent to which the presence of mannan can hamper the access of cellulases to cellulose. Several studies by Beukes et al. (2008) and Beukes and Pletschke (2010, 2011) have investigated combinations of hemicellulases with cellulases and the associated synergy. Beukes et al. (2008) found that there were synergistic relationships between EngE, XynA and ManA and that the highest degree of synergy on sugarcane bagasse was found when the ratio of EngE:XynA:ManA was 25:25:50 (Beukes et al. 2008). This provides a glimpse into the important role that xylanases and mannanases may play in the degradation of complex substrates, and this is further evaluated here.

## Materials and methods

### Expression and purification of enzymes

Cloned genes of *engE* (Tamaru and Doi 1999; AF105331), *manA* (Tamaru and Doi 2000; AF132735) and *xynA* (Kosugi et al. 2002; AY604045) were kindly donated by Prof. Roy Doi (University of California Davis, CA, USA). Three cultures of *Escherichia coli* BL21 (DE3) cells, transformed with the recombinant plasmids pET29-engA, pET29b-manA and pET29b-xynA, were expressed and purified using affinity chromatography as described in Beukes and Pletschke (2010). However, induction was carried out at 37 °C and induction periods of 1 h (EngE), 4 h (XynA) and 3 h (ManA) were used for optimum expression. The efficiency of the purification was determined by electrophoresis of purified protein samples on a 12% reducing SDS-PAGE gel (Laemmli 1970). The activity of the purified protein samples were confirmed using commercial substrates as described below.

### Protein assay

The protein concentration was determined using the Bradford protein assay (Bradford 1976) with bovine serum albumin (BSA) as a standard according to the protocol supplied by Sigma for Bradford’s reagent. The protein concentrations together with the molecular weight of each purified enzyme were used to calculate the molar concentration for synergy studies.

### Enzyme activity assay

The activities of the enzymes were determined using a modified dinitrosalicylic acid (DNS) assay (Miller 1959). The assay consisted of 50 μl of the enzyme with 250 μl of 50 mM citrate buffer (pH 5) in the presence of 100 μl of a 2% (w/v) concentration of the appropriate substrate, namely low viscosity carboxymethylcellulose (CMC), locust bean gum (LBG) and birchwood xylan (BWX) for EngE, ManA and XynA, respectively. Assays were carried out at 50 °C for 30 min on commercial substrates. After incubation, the assay mixture was microfuged at 16,000×*g* for 1 min, and 150 μl of the supernatant was added to 300 μl of DNS reagent. The color reaction was developed for 5 min at 100 °C and terminated with 5 min incubation on ice. The absorbance was measured at 540 nm, using a Powerwave_x_ microtiterplate reader (Bio-tek Instruments). Activity was calculated as glucose equivalents, and 1 U was defined as the amount of enzyme required to release 1 μmol of reducing sugar per minute under the assay conditions.

### Synergy studies

Synergy studies were carried out on three complex substrates, namely corn stalk (*Zea mays*), kikuyu grass (*Pennisetum clandestinum*) and pineapple fruit pulp. The complex substrates were homogenized in a blender to an even consistency of small particles and then washed, dried and autoclaved before use in the assays, with the exception of the pineapple fruit pulp which was not dried. The synergistic relationships between the purified enzymes were determined by combining two enzymes in varying molar ratios (as a percentage of the total) (100:0, 75:25, 50:50, 25:75, 0:100) and carrying out assays as described above The assays with the complex substrates were incubated at 50 °C for 7 days. All assays were performed in triplicate. The degree of synergy was calculated based on the specific activity of combinations, divided by the sum or the activity of the enzymes acting individually on the substrate.

## Results

The different enzymes were each purified to a single band using SDS-PAGE, and all purified enzymes displayed activity on their respective substrates (data not shown).

**Fig. 1:**
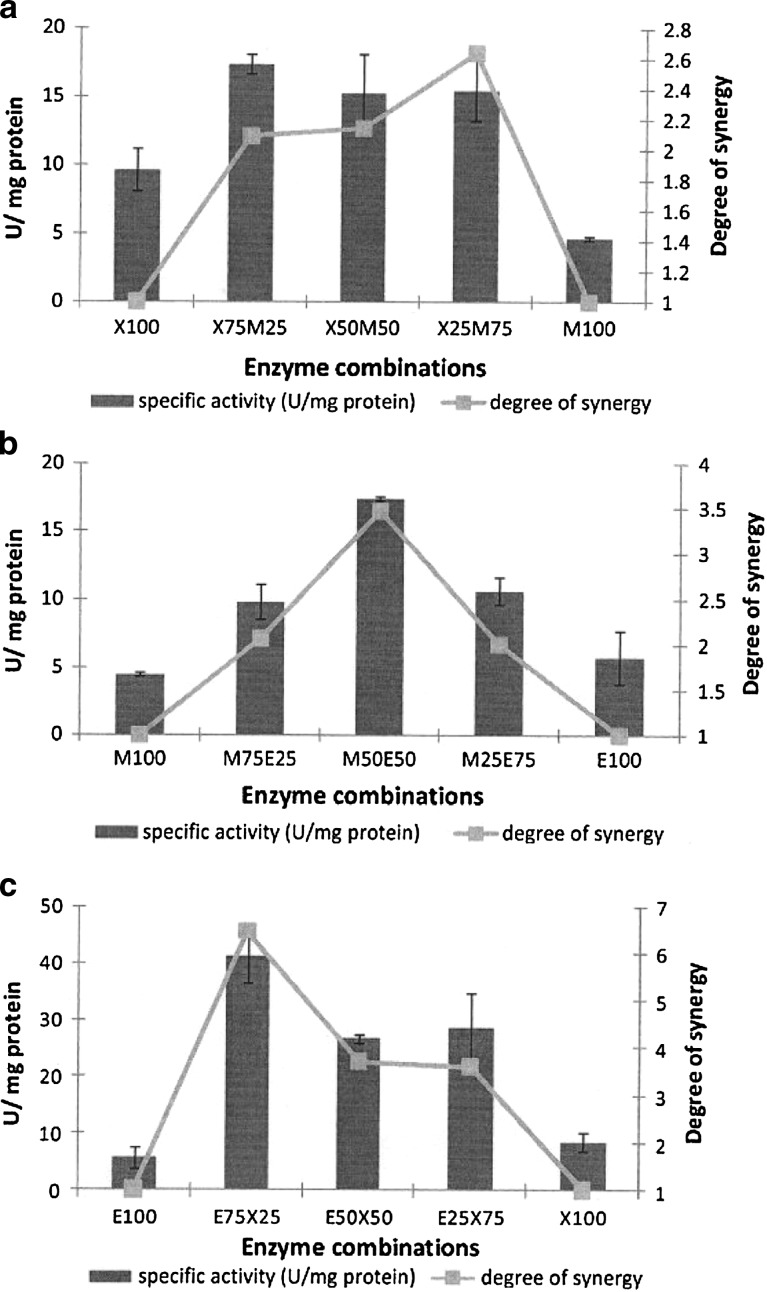
Synergy graphs using the complex substrate, corn stalk. **a** XynA:ManA enzyme combination. **b** ManA:EngE enzyme combination. **c** EngE:XynA enzyme combination. Values are presented as mean values ± SD (*n* = 3)

Table 1 summarizes the ratios that achieved the optimal degree of synergy for each substrate, together with the specific activity of that ratio. Figure 1 illustrates the results of the different enzyme combinations on corn stalk. The results for corn stalk indicated that the optimum degree of synergy was achieved at a ratio of XynA (25%):ManA (75%) (Fig. 1a). However, the optimal specific activity was found at a ratio of XynA (75%):ManA (25%). The ManA:EngE enzyme combination (Fig. 1b) revealed that the specific activity and degree of synergy both peaked at a ManA (50%):EngE (50%) ratio. The EngE:XynA combination (Fig. 1c) reached a maximum specific activity and degree of synergy at EngE (75%):XynA (25%). This combination of enzymes resulted in the highest specific activity and degree of synergy with corn stalk, with values of 41.1 U/mg protein and 6.3, respectively. This was the highest degree of synergy found with any combination on any substrate.

**Table 1:** Summary of the results obtained from synergy studies, showing the enzyme combinations where optimal degree of synergy was observed and the specific activity associated with that combination ± SD (*n* = 3)

Substrates	Enzyme combinations	Ratios (%)	Activity (U/mg protein) ± SD	Degree of synergy ± SD
Corn stalk	XynA:ManA	X25:M75	15.5 ± 2.2	2.6 ± 0.3
	ManA:EngE	M50:E50	17.4 ± 0.1	3.5 ± 0.6
	EngE:XynA	E75:X25	41.1 ± 4.6	6.5 ± 1.7
Grass	XynA:ManA	X25:M75	14.6 ± 0.9	1.2 ± 0.1
	ManA:EngE	M25:E75	29.5 ± 9.3	2.0 ± 0.6
	EngE:XynA	E75:X25	44.1 ± 0.9	3.3 ± 0.2
Pineapple fruit pulp	XynA:ManA	X25:M75	39.9 ± 7.2	2.5 ± 0.7
	ManA:EngE	M50:E50	52.4 ± 9.8	2.8 ± 1.1
	EngE:XynA	E50:X50	43.4 ± 4.5	2.4 ± 0.5

**Fig. 2:**
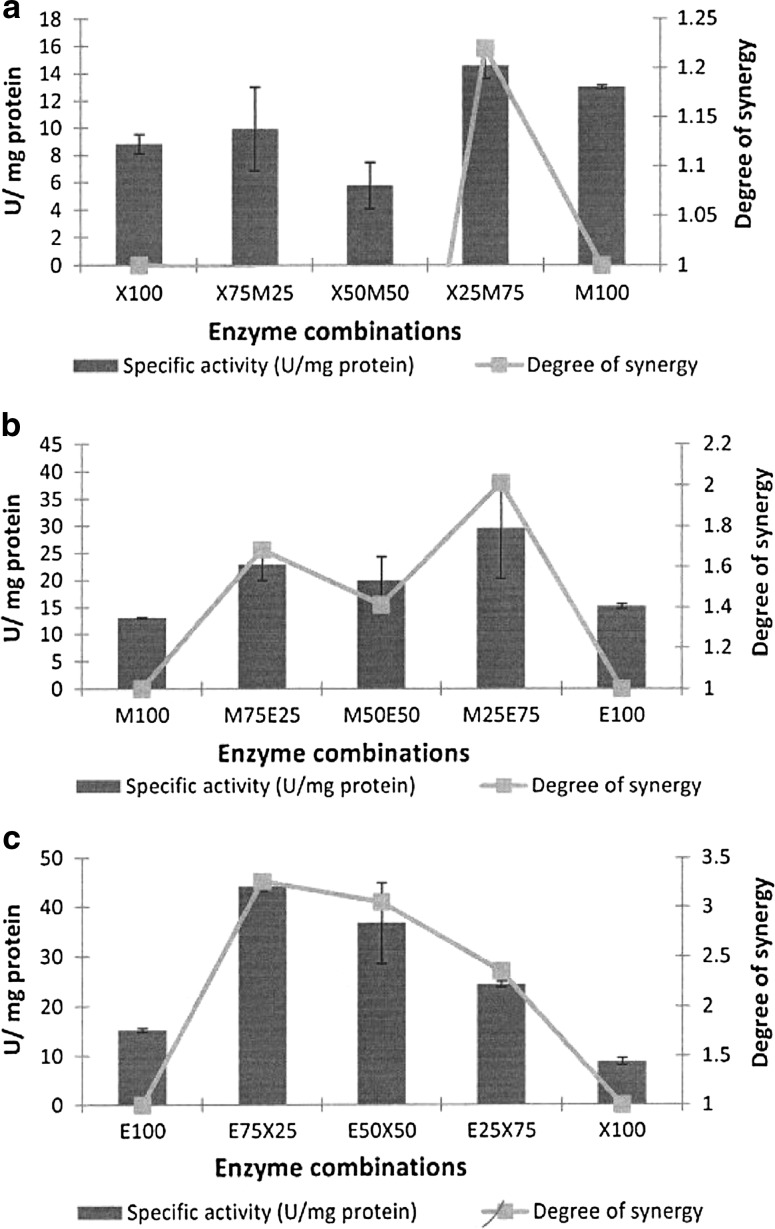
Synergy graphs using the complex substrate, milled grass. **a** XynA:ManA enzyme combination. **b** ManA:EngE enzyme combination. **c** EngE:XynA enzyme combination. Values are presented as mean values ± SD (*n* = 3)

The grass was the second complex substrate on which synergy between enzymes was investigated (Fig. 2). Figure 2a shows that the XynA:ManA enzyme combination had a maximum specific activity at a ratio of XynA (25%):ManA (75%) which corresponded to the optimum degree of synergy of 1.2 at this ratio. The highest specific activity and degree of synergy for the ManA:EngE combination with the grass were found to be at a ratio of ManA (25%):EngE (75%) (Fig. 2b), with specific activity measured at 29.5 U/mg protein and the degree of synergy at 2.0. The EngE:XynA combination indicated that both the specific activity and degree of synergy were optimal at a ratio of EngE (75%):XynA (25%) (Fig. 2c), similar to that found with the corn stalk. The specific activity was 44.1 U/mg protein and the degree of synergy 3.3.

**Fig. 3:**
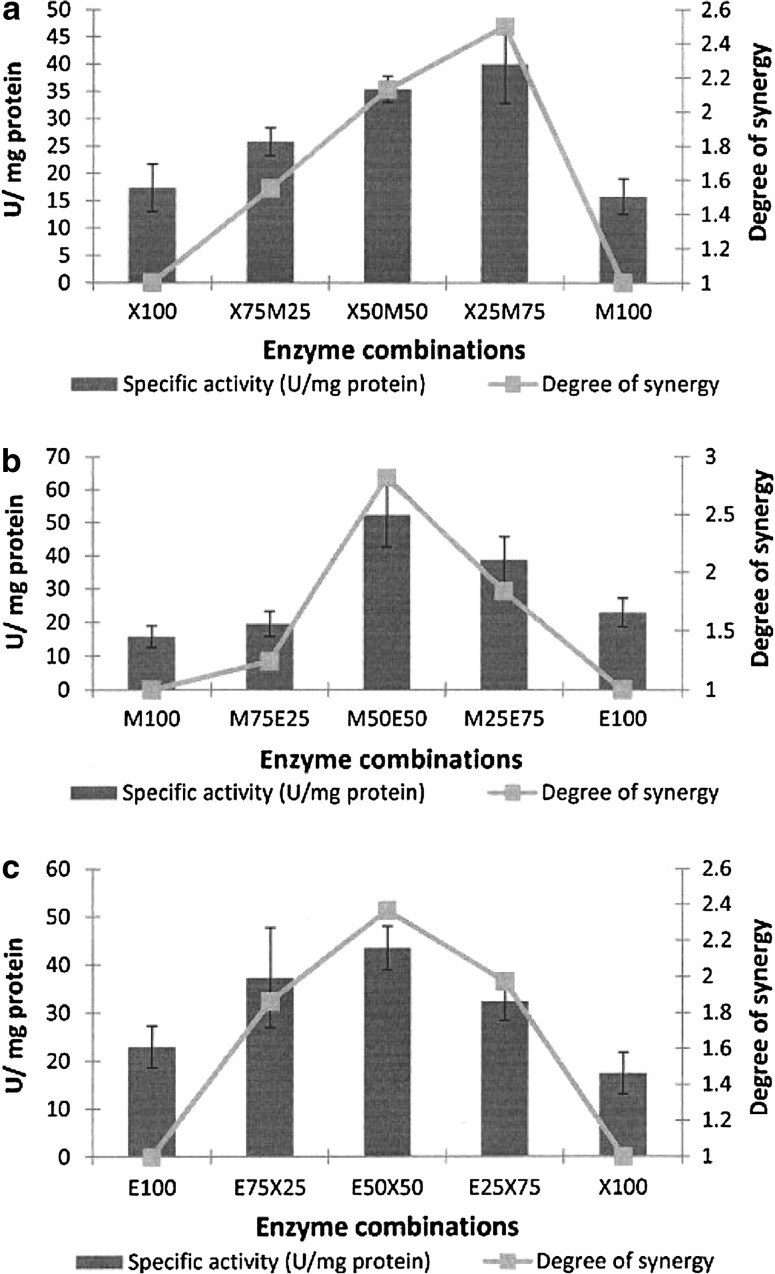
Synergy graphs using the complex substrate, pineapple fruit pulp. **a** XynA:ManA enzyme combination. **b** ManA:EngE enzyme combination. **c** EngE:XynA enzyme combination. Values are presented as mean values ± SD (*n* = 3)

The pineapple fruit pulp responded differently to enzymatic degradation and displayed the highest overall hydrolysis with specific activity ranging from 40 to 52 U/mg protein (Fig. 3; Table 1). The XynA:ManA enzyme combination resulted in a maximum specific activity (40 U/mg protein) and degree of synergy (2.5) at a ratio of XynA (25%):ManA (75%) (Fig. 3a). The other two combinations of enzymes, ManA:EngE (Fig. 3b) and EngE:XynA (Fig. 3c), both had maximal specific activity and degrees of synergy at enzyme ratios of 50:50%. The ManA (50%):EngE (50%) had the highest overall activity of 52.4 U/mg protein with a degree of synergy of 2.8, while the EngE (50%):XynA (50%) had a specific activity of 43.4 U/mg protein and a degree of synergy of 2.4.

A summary of the data obtained in the synergy studies on complex substrates (Table 1) indicated that all the enzyme combinations on all the substrates displayed a synergistic relationship although some were much higher than others. The highest specific activities and degrees of synergy were found in enzyme combinations containing EngE. Apart from one combination (XynA (75%):ManA (25%), it was observed that the highest levels of activity (and therefore degree of hydrolysis) of the enzyme combinations correlated with the optimal degrees of synergy, indicating that increased synergy resulted in higher sugar release from substrates.

## Discussion

A measurement of the degree of synergy between enzymes allows for the determination of areas of recalcitrance and the manner in which enzymes can cooperate to overcome this recalcitrance. According to Banerjee et al. (2010), the ratio of an enzyme required is based on the linkage it cleaves and the occurence of that linkage within the structure of the substrate. It has been established from synergy studies that lignin and hemicellulose both hamper access of cellulases to cellulose (Varnai et al. 2010). Lignin is generally removed or disrupted through various chemical pretreatments (Hendriks and Zeeman 2009; Sun and Cheng 2002). In this study, a simple physical pretreatment step of homogenization was included as it has been demonstrated that such physical disruption of cells contributes to hydrolysis through an increase of surface area and a reduction in the degree of polymerization (Hendriks and Zeeman 2009; Jung et al. 2000; Walker and Wilson 1991).

It is generally accepted that hemicellulose is associated with cellulose through hydrogen bonding (Beg et al. 2001), and therefore, it is has been argued that addition of xylanase will improve cellulose degradation (Garcia-Aparicio et al. 2007; Kumar and Wyman 2009; Merino and Cherry 2007; Selig et al. 2009). Garcia-Aparicio et al. (2007) found that, even where the majority of xylan was removed through pretreatment steps, xylanase supplementation still enhanced hydrolysis of the substrate. However, based on the results of this study, we would argue that perhaps the presence of low levels of mannan in substrates may hamper the access of cellulases in a similar manner which has not been investigated. The fact that combinations of EngE or XynA display a synergy in combination with ManA indicate that degradation of mannan in the substrate contributed to the activity of the other enzymes. The mannan composition of substrates is often not ascertained even though mannan-containing polysaccharides are widely distributed in cell walls of plants (Hrmova et al. 2006). Mannan is present to a minor extent in the cell walls of cereals and grasses of the Poaceae family (Hrmova et al. 2006), but in wood, glucomannans constitute 3–5% of hardwoods and galactoglucomannan 15–25% of softwoods (Hägglund 2002). Therefore, it is apparent that many lignocellulose substrates contain some level of mannan and, according to Carpita (1996), it is tightly bound to cellulose and affects the accessibility of cellulases to cellulose.

The corn stalk and grass substrates displayed maximal activity and degrees of synergy with the EngE:XynA combination in comparison to the EngE:ManA combination. This is potentially due to the fact that the substrates have a higher content of xylan to mannan (Foyle et al. 2007; Murashima et al*.*^2003^). The pineapple fruit pulp displayed optimum activity and degree of synergy with a ManA:EngE combination, indicating that mannan was a main hemicellulose constituent in the pineapple fruit pulp.

This study demonstrated that the cellulosomal enzymes EngE, ManA and XynA of *C. cellulovorans* have a synergistic effect on each other, that the relative amount of each enzyme affects the degree of synergy, and that the percentage of the enzymes that is required to obtain the highest degree of synergy may depend on the substrate. The variation of the enzyme ratios in the optimal combinations is probably related to the chemical composition of each substrate. In this study, the enzyme combinations that resulted in the highest specific activity and degree of synergy on each of the three substrates contained EngE as one of the enzymes since cellulose is the largest component present. Therefore, endoglucanases are essential for the efficient digestion of biomass as a large percentage of plant matter is cellulosic. The hemicellulases required, in turn, are dependent on the hemicellulosic composition of the substrate. But the synergistic effects found with ManA suggest that levels of mannan are present in these substrates and that mannanases may be an important addition to enzyme consortia for the degradation of complex substrates.

This study provides an insight into the design of artificial mini-cellulosomes and suitable enzyme cocktails for feedstock hydrolysis and shows that certain combinations of enzymes are better suited to certain substrates. Analysis of the composition of substrates is therefore crucial. However, even where very low hemicellulose levels are present, it is becoming apparent that hemicellulase supplementation is still essential for effective degradation of substrates.

In conclusion, it is evident that the investigation of synergism between enzymes and different substrates will play a vital role in maximizing and optimizing the degradation of biomass waste products to fermentable sugars for subsequent biofuel production.

## References

[CR1] Andersen N, Johansen KS, Michelsen M, Stenby EH, Krogh KBRM, Olsson L (2008). Hydrolysis of cellulose using mono-component enzymes shows synergy during hydrolysis of phosphoric acid swollen cellulose (PASC), but competition on Avicel. Enzym Microb Technol.

[CR2] Banerjee G, Scott-Craig JS, Walton JD (2010). Improving enzymes for biomass conversion: a basic research perspective. Bioenerg Res.

[CR3] Ban-Koffi L, Han YW (1990). Alcohol production from pineapple waste. World J Microbiol Biotechnol.

[CR4] Bayer EA, Lamed R, Himmel ME (2007). The potential of cellulases and cellulosomes for cellulosic waste management. Curr Opin Biotechnol.

[CR5] Beg QK, Kapoor M, Mahajan L, Hoondal GS (2001). Microbial xylanases and their industrial applications: a review. Appl Microbiol Biotechnol.

[CR6] Beukes N, Pletschke BI (2010). Effect of lime pre-treatment on the synergistic hydrolysis of sugarcane bagasse by hemicellulases. Bioresour Technol.

[CR7] Beukes N, Pletschke BI (2011). Effect of alkaline pre-treatment on enzyme synergy for efficient hemicellulose hydrolysis in sugarcane bagasse. Bioresour Technol.

[CR8] Beukes N, Chan H, Doi RH, Pletschke BI (2008). Synergistic associations between *Clostridium cellulovorans* enzymes XynA, ManA and EngE against sugarcane bagasse. Enzyme Microb Technol.

[CR9] Boisset C, Petrequin C, Chanzy H, Henrissat B, Schulein M (2001). Optimized mixtures of recombinant *Humicola insolens* cellulases for the biodegradation of crystalline cellulose. Biotechnol Bioeng.

[CR10] Bradford MM (1976). A rapid and sensitive method for the quantitation of microgram quantities of protein utilizing the principle of protein-dye binding. Anal Biochem.

[CR11] Carpita NC (1996). Structure and biogenesis of the cell walls in grasses. Ann Rev Plant Phys Plant Mol Biol.

[CR12] Dijkerman R, Bhansing DCP, Op den Camp HJM, van der Drift C, Vogels GD (1997). Degradation of structural polysaccharides by the plant cell-wall degrading enzyme system from anaerobic fungi: an application study. Enzym Microb Technol.

[CR13] Foyle T, Jennings L, Mulcahy P (2007). Compositional analysis of lignocellulosic materials: evaluation of methods used for sugar analysis of wastepaper and straw. Bioresour Technol.

[CR14] Gao D, Chundawat SPS, Krishnan C, Balan V, Dale BE (2010). Mixture optimization of six core glycosyl hydrolases for maximizing saccharification of ammonia fiber expansion (AFEX) pretreated corn stover. Bioresour Technol.

[CR15] Garcia-Aparicio MP, Ballesteros M, Manzanares P, Ballesteros I, Gonzalez A, Negro MJ (2007). Xylanase contribution to the efficiency of cellulose enzymatic hydrolysis of barley straw. Appl Biochem Biotechnol.

[CR16] Hägglund P (2002). Mannan-hydrolysis by hemicellulases.

[CR17] Hendriks ATWM, Zeeman G (2009). Pretreatments to enhance the digestibility of lignocellulosic biomass. Bioresour Technol.

[CR18] Himmel ME, Ding S-Y, Johnson DK, Adney WS, Nimlos MR, Brady JW, Foust TD (2007). Biomass recalcitrance: engineering plants and enzymes for biofuel production. Science.

[CR19] Hrmova M, Burton RA, Biely P, Lahnstein J, Fincher GB (2006) Hydrolysis of (1,4)-β-d-mannans in barley (*Hordeum vulgare* L.) is mediated by the concerted action of (1,4)-β-d-mannan endohydrolase and β-d-mannosidase. Biochem J 399:77–90 10.1042/BJ20060170 PMC1570163 16771710

[CR20] Jung H-JG, Jorgensen MA, Linn JG, Engels FM (2000). Impact of accessibility and chemical composition on cell wall polysaccharide degradability of maize and lucerne stems. J Sci Food Agric.

[CR21] Kosugi A, Murashima K, Doi RH (2002). Xylanase and acetyl xylan esterase activities of XynA, a key subunit of the *Clostridium cellulovorans* cellulosome for xylan degradation. Appl Environ Microbiol.

[CR22] Kumar R, Wyman CE (2009). Effects of cellulase and xylanase enzymes on the deconstruction of solids from pretreatment of poplar by leading technologies. Biotechnol Prog.

[CR23] Laemmli UK (1970). Cleavage of structural proteins during the assemble of the head of the bacteriophage T4. Nature.

[CR24] Li H, Kim N-J, Jiang M, Kang JW, Chang HN (2009). Simultaneous saccharification and fermentation of lignocellulosic residues pretreated with phosphoric acid-acetone for bioethanol production. Bioresour Technol.

[CR25] Lynd LR, Cushman JH, Nichols RJ, Wyman CE (1991). Fuel ethanol from cellulosic biomass. Science.

[CR26] Merino ST, Cherry J (2007). Progress and challenges in enzyme development for biomass utilization. Adv Biochem Engin/Biotechnol.

[CR27] Miller GL (1959). Use of dinitrosalicyclic acid reagent for the determination of reducing sugars. Anal Chem.

[CR28] Murashima K, Kosugi A, Doi RH (2003). Synergistic effects of cellulosomal xylanase and cellulases from *Clostridium cellulovorans* on plant cell wall degradation. J Bacteriol.

[CR29] Prior BA, Day DF (2008). Hydrolysis of ammonia-pretreated sugarcane bagasse with cellulase, β-glucosidase, and hemicellulase preparations. Appl Biochem Biotechnol.

[CR30] Selig MJ, Adney WS, Himmel ME, Decker SR (2009). The impact of cell wall acetylation on corn stover hydrolysis by cellulolytic and xylanolytic enzymes. Cellulose.

[CR31] Sun Y, Cheng J (2002). Hydrolysis of lignocellulosic materials for ethanol production: a review. Bioresour Technol.

[CR32] Tamaru Y, Doi RH (1999). Three surface layer homology domains at the N-terminus of the *Clostridium cellulovorans* major cellulosomal subunit, EngE. J Bacteriol.

[CR33] Tamaru Y, Doi RH (2000). The *engL* gene cluster of *Clostridium cellulovorans* contains a gene for cellulosomal ManA. J Bacteriol.

[CR34] Teeri TT (1997). Crystalline cellulose degradation: new insight into the function of cellobiohydrolases. Trends Biotechnol.

[CR35] Varnai A, Siika-aho M, Viikari L (2010). Restriction of the enzymatic hydrolysis of steam-pretreated spruce by lignin and hemicellulose. Enzym Microb Technol.

[CR36] Walker LP, Wilson DB (1991). Enzymatic hydrolysis of cellulose: an overview. Bioresour Technol.

[CR37] Woodward J (1991). Synergism in cellulase systems. Bioresour Technol.

